# Novel Mixed-Matrix Pervaporation Membrane Based on Polyether Block Amide Modified with Ho-Based Metal–Organic Framework

**DOI:** 10.3390/polym16233245

**Published:** 2024-11-22

**Authors:** Anna Kuzminova, Mariia Dmitrenko, Anastasia Stepanova, Anna Karyakina, Artem Selyutin, Rongxin Su, Anastasia Penkova

**Affiliations:** 1 St. Petersburg State University, 7/9 Universitetskaya Nab., St. Petersburg 199034, Russia; m.dmitrienko@spbu.ru (M.D.); st113221@student.spbu.ru (A.S.); yara_2000@mail.ru (A.K.); a.selyutin@spbu.ru (A.S.); 2 State Key Laboratory of Chemical Engineering, School of Chemical Engineering and Technology, Tianjin University, Tianjin 300072, China; surx@tju.edu.cn

**Keywords:** polyether block amide, metal–organic frameworks, mixed-matrix membranes, isopropanol, phenol, pervaporation

## Abstract

Segmented polymers, such as polyether block amide (PEBA), exhibit unique properties due to the combination of different segments. PEBA consists of soft polyester and rigid polyamide blocks, enabling its use in various industrial applications, including membrane technologies. In this study, PEBA membranes modified with a holmium-based metal–organic framework (Ho-1,3,5-H_3_btc) were developed for enhanced pervaporation separation of water/isopropanol and water/phenol mixtures. The effect of 1–7 wt.% Ho-1,3,5-H_3_btc content variation and the selection of a porous substrate (commercial from fluoroplast F42L (MFFC) and developed membranes from polyvinylidene fluoride without (PVDF) and with a non-woven polyester support (PVDF-s)) on dense and/or supported membrane properties, respectively, was investigated. The dense and supported PEBA/Ho-1,3,5-H_3_btc membranes were studied by use of Fourier transform infrared spectroscopy, scanning electron and atomic force microscopies, swelling measurements, and pervaporation experiments. The supported membrane from PEBA with 5 wt.% Ho-1,3,5-H_3_btc applied onto the PVDF-s substrate exhibited optimal pervaporation performance: a 1040 g/(m^2^h) permeation flux and a 5.2 separation factor in water/phenol (1 wt.%) mixture separation at 50 °C due to optimal values of roughness, swelling degree, and selective layer thickness. This finding highlights the potential of incorporating Ho-1,3,5-H_3_btc into PEBA for developing high-performance pervaporation membranes.

## 1. Introduction

Polymers, with their unique properties and diverse applications, play a key role in the modern world and industries, including construction, automotive, medical, electronics, packaging, and textiles [1]. Among polymers, segmented polymers deserve special attention, as they consist of several segments with different properties. This structure makes it possible to obtain materials with combined unique properties that cannot be achieved with homopolymers [2,3].

Polyether block amides (PEBAs) or Pebax^®^ elastomers (trade name) are a class of block copolymers that combine the unique structural properties of rigid polyamide blocks, soft polyester blocks, and unplasticized thermoplastic elastomers (TPEs) [4]. Rigid polyamide blocks provide mechanical strength and thermal stability, while soft polyester blocks impart flexibility and elasticity, making them promising for use in membrane technologies. PEBA-based membranes have already proven themselves as membrane materials for use in reverse osmosis, dialysis, gas separation, and pervaporation [5,6]. However, the transport characteristics of the currently available PEBA membranes are insufficient for the effective removal of organics from water. PEBA membranes are actively used for pervaporation separation of different mixtures, such as pyridine/water [7,8,9], ethanol/water [10,11,12,13], toluene/water [14,15], aniline/water [16,17], phenol/water [16,18,19,20,21,22], furfural/water [23], ethyl acetate/water [24,25,26], isopropylbenzene/water [27], 1-ethyl-2-methylbenzene/water [28], butanol/water [29,30], isopropanol/water [31,32,33], and propyl propionate/water [34]. PEBA-based membranes are most often used to remove phenol from water. Phenolic compounds can be potentially toxic, carcinogenic, teratogenic, and mutagenic. The separation and recovery of phenolic compounds are crucial from both an industrial perspective and in terms of environmental safety. Conventional methods such as extraction, distillation, chemical oxidation, electrochemical oxidation, and adsorption can successfully remove phenolic compounds from water [35,36,37,38]. However, these methods have a number of disadvantages, such as the use of a large number of chemical reagents, high energy consumption and costs, etc. [39]. Some advanced technologies are free of these disadvantages, for example, the use of various enzymes (peroxidase, laccase, and tyrosinase) [40] allows for the removal of phenolic compounds under mild conditions, but there is a problem with further processing of the enzyme medium. Membrane technologies, on the other hand, do not have these drawbacks, and the creation of membrane modules using cascading purification steps has great potential for industrial applications of purification. Alcohols also contain a hydroxyl group in their structure such as phenols, and the removal of alcohols, particularly isopropanol, is a pressing issue due to its impact on environmental safety, human health, and water quality. In contrast to traditional processes, pervaporation demonstrates significant potential in the removal of organic compounds, offering high separation efficiency, simple equipment, ease of operation, and low energy consumption. This technology is considered a promising solution for separating and recovering low-concentration phenols from wastewater.

There are several studies presented in the literature on the creation of mixed-matrix membranes (MMMs) based on PEBAs to enhance the pervaporation process. Different substances such as 4-(trifluoromethyl)-N(pyridine-2-yl)benzamide and 4-(dimethylamino)-N(pyridine-2-yl)benzamide [12], mesoporous silicate MCM-41 [15,41], two-dimensional molybdenum disulfide nanosheets [8], NaX nanozeolites [42], graphene oxide (GO) modified with ionic liquid (IL) (N-octylpyridiniunm bis (trifluoromethyl) sulfonyl imide [OPY][Tf2N]) [30], zeolitic metal azolate frameworks, RHO-[Zn(Heim)_2_] (MAF-6, Heim = 2-ethylimidazole) [22], mesoporous molecular sieves MCM-41 modified with IL (1-ethyl-3-vinylimidazolium bis[(trifluoromethyl)sulfonyl]imide ([EVIM][Tf_2_N]) and N-octyl-pyridinium bis[(trifluoromethyl)sulfonyl]imide ([OMPY][Tf2N])) [29], ZSM-5 zeolite [26,43,44] and [Hmim][PF6] IL [25], Cu_2_O nanocrystals [9], carbon nanotubes [45], metal–organic frameworks (MOFs) [7,11,13,16,18,20,21,23,24,46,47,48,49], etc., have been used as modifiers for PEBA membranes.

MOFs are very relevant and promising modifiers for PEBA-based membranes due to their unique properties. In [46], the use of zeolitic imidazolate framework-71 (ZIF-71) nanoparticles and nanosheets as fillers in PEBA membranes was studied for efficient phenol–water separation. The results showed that the incorporation of ZIF-71 nanosheets led to improved selectivity, mainly due to the formation of a “brick-and-mortar” structure within the membrane that inhibited water transport while maintaining phenol permeation. MMMs from PEBAs were developed by incorporating ZIF-8 nanoparticles using interface induction to control their directional distribution [47]. This controlled distribution improved the stability and separation performance of the membranes. The directional distribution of the nanoparticles protected the ZIF-8 structure from direct contact with the feed, ensuring long-term stability over 100 h of operation. Reference [48] focused on overcoming the permeability–selectivity trade-off in pervaporation by designing high-performance PEBA MMMs. A novel solvent-assisted linker exchange strategy was employed to introduce fluoroalkyl groups into MOF-808, tailoring its pore size. The resulting fluorinated MOF-808/PEBA MMMs exhibited enhanced butanol permeation and separation performance due to the “push–pull effect” created by the fluorinated MOF-808, enhancing butanol affinity and repelling water. The use of functionalized MIL-101 within a PEBA matrix was studied to create MMMs for the selective separation of phenol [49]. By introducing different organic ligands to MIL-101, improved interfacial compatibility and hydrophobicity compared to unmodified MIL-101 were demonstrated, leading to improved performance in phenol pervaporation. PEBA/ZIF-71 membranes were developed for the pervaporation removal of phenol and aniline from water [16]. The incorporation of ZIF-71 resulted in a significant reduction in water membrane permeability, maintaining or slightly reducing the permeability of phenol and aniline, causing increased selectivity toward these aromatic compounds due to the preferential sorption and diffusion of the aromatic solutes within the inner ZIF-71 channels. In [18], PEBA membranes were modified with porous HZIF-8 (ZIF-8 with polystyrene (PS) to improve compatibility. The resulting PEBA/HZIF-8 membrane exhibited enhanced phenol/water separation performance due to the π–π interaction between the imidazole ring skeleton in HZIF-8 and phenol molecules. This interaction was facilitated by the PS on the surface of HZIF-8, leading to improved compatibility between the filler and the PEBA matrix.

Thus, the introduction of MOFs into the PEBA matrix led to an improvement in the transport characteristics of MMMs. To the best of our knowledge, there is no research devoted to the development of pervaporation MMMs based on PEBAs modified with Ho-based MOFs. This work is a continuation of a previous work [50], where a range of five Ho-based MOFs (Ho-1,3,5-H_3_btc, Ho-1,2,4-H_3_btc, Ho-1,2-H_2_bdc, Ho-1,3-H_2_bdc, Ho-1,4-H_2_bdc) were synthesized and studied as perspective modifiers for the PEBA matrix. The developed Ho-1,3,5-H_3_btc turned out to be the most promising for PEBA modification in vacuum filtration for dye removal due to its needle-shaped structure, crystal morphology, and uniform distribution of particles in the polymer matrix. However, its effect as a modifier of pervaporation PEBA membranes has not yet been studied.

Thus, the aim of this study was to study the effect of Ho-1,3,5-H_3_btc on the structural, physicochemical, and transport properties of PEBA-based membranes and to develop pervaporation PEBA/Ho-1,3,5-H_3_btc membranes with improved performance for enhanced isopropanol and phenol removal from water. The effect of Ho-1,3,5-H_3_btc content variation and the selection of a porous substrate (commercial MFFC and developed substrate from PVDF without/with the use of a non-woven polyester support) on dense and supported membrane properties, respectively, was investigated. The study of developed MMMs was carried out by methods of Fourier transform infrared spectroscopy, scanning electron microscopy, atomic force microscopy, and swelling measurements. The transport properties of developed membranes were evaluated for pervaporation separation of water/isopropanol and water/phenol mixtures.

## 2. Materials and Methods

### 2.1. Materials

Polyester block amide (PEBA, Pebax-2533) from Hebei Luozheng Technology Co., Ltd. (Shijiazhuang, China) was used as a membrane material. An Ho-based MOF—Ho-1,3,5-H_3_btc (prepared from Ho(NO_3_)_3_·5H_2_O and trimesic acid)—was used as a membrane modifier. The synthesis and characterization of highly porous Ho-1,3,5-H_3_btc (with needle-shaped structure, a needle length of ~50 µm, and crystal morphology) was described in our previous study [50]. Isopropanol (i-PrOH), N,N′-dimethylacetamide (DMA), phenol (PhOH), and n-butanol from Vekton (St. Petersburg, Russia) were used without further purification. Polyvinylidene fluoride (PVDF, XF2170P, molecular weight of 300,000–500,000 g/mol) from Transcool LLC (Moscow, Russia) was used as a polymer for porous substrate preparation onto a non-woven polyester support (Novatexx 2430, Freudenberg Filtration Technologies, Weinheim, Germany). A commercial membrane MFFC (based on fluoroplast F42L) from Vladipor (Vladimir, Russia) was also used as a porous substrate.

### 2.2. Dense Membrane Preparation

To develop dense membranes, a 10 wt.% PEBA solution and PEBA/Ho-1,3,5-H_3_btc composites were prepared in n-butanol at 85 °C for 5 h with constant stirring. Up to 7 wt.% Ho-1,3,5-H_3_btc with respect to the PEBA weight was added into the polymer matrix. Then, PEBA and PEBA/ Ho-1,3,5-H_3_btc dispersions were sonicated at ambient temperature, poured into glass Petri dishes for the formation of dense membranes by solvent evaporation, and placed at 60 °C in an oven for 24 h. The thickness of the dense PEBA and PEBA/ Ho-1,3,5-H_3_btc membranes measured with a micrometer was equal to 80 ± 10 μm.

### 2.3. Supported Membrane Preparation

The preparation of the supported PEBA and PEBA/Ho-1,3,5-H_3_btc membranes was carried out as follows: the prepared PEBA or PEBA/ Ho-1,3,5-H_3_btc dispersions were applied onto a porous PVDF-based or commercial MFFC membrane followed by drying in air for 24 h. The concentration of the PEBA solution was 3 wt.%.

To prepare a porous PVDF substrate, 15 wt.% PVDF was dissolved in DMA at 100–120 °C for 4 h with constant stirring using an overhead stirrer. Two types of porous PVDF substrate were prepared by using non-solvent-induced phase separation (NIPS): without (PVDF substrate) and with a non-woven polyester support (PVDF-s substrate). The PVDF solution was deposited onto a glass or a non-woven polyester support using a casting blade with a gap width of 200 µm, followed by immersion in a coagulation bath containing distilled water at ambient temperature [51].

Table 1 shows the designations of the membranes developed in this study.

### 2.4. Pervaporation Experiment

The transport properties of the developed PEBA and PEBA/Ho-1,3,5-H_3_btc membranes were studied for pervaporation using a laboratory cell with stirring at 22 °C [52]. The compositions of the feed and permeate were studied on a gas chromatograph, Chromatec Crystal 5000.2 from Chromatec (Nizhny Novgorod, Russia), with a column “Hayesep R” and a thermal conductivity detector.

The permeation flux *J* (kg/(m^2^h)) of the developed PEBA and PEBA/Ho-1,3,5-H_3_btc was calculated by Equation (1) [53]:

(1) $$J=\frac{W}{A\cdot t},$$

where *W* (kg) is the weight of the permeate (the mixture that permeated through the membrane), *A* (m^2^) is the effective membrane area, and *t* (h) is the time of the measurement.

The separation factor (*β*) was calculated by Equation (2) [54]:

(2) β=yiyjxixj,

where *y_i_* and *y_j_* are the weight of the components—isopropanol or phenol and water in the permeate; *x_i_* and *x_j_* are the weight of the components—isopropanol or phenol and water in the feed.

The permeance *P*/*l* was calculated by Equation (3) [48]:

(3) $$P/l=\frac{j_{i}}{p_{i_{f}}-p_{i_{p}}},$$

where *j_i_* is the partial flux of component *i*, l is the membrane’s thickness, and $p_{i_{f}}$ and $p_{i_{p}}$ are the vapor pressures of component *i* in the feed and the permeate, respectively. Gas permeation units (GPUs) were used to express the permeances of isopropanol and water (1 GPU = 1 × 10^−6^ cm^3^ (STP)/cm^2^ s cm Hg; 1 m^3^ m/m^2^ s kPa = 1.33 × 10^8^ GPU).

The pervaporation separation index (PSI) was calculated by Equation (4) [55]:

(4) $$PSI=J\cdot \left(\beta -1\right),$$

To ensure the reliability of the results, all data were collected in triplicate, and the average value was used for analysis. The average accuracies obtained were as follows: ±0.7% for iPrOH and PhOH content in the permeate, ±5% for permeation flux of the dense membranes, and ±8% for permeation flux of the supported membranes.

### 2.5. Fourier Transform Infrared Spectroscopy

The structural analysis of dense PEBA and PEBA/Ho-1,3,5-H_3_btc membranes was performed using a BRUKER-TENSOR 27 Spectrometer (Bruker, Ettlingen, Germany) equipped with an attenuated total reflectance (ATR) accessory. The spectra were collected in the range of 600–4000 cm^−1^ at ambient temperature.

### 2.6. Atomic Force Microscopy

The surface topography of the PEBA and PEBA/Ho-1,3,5-H_3_btc membranes was studied by atomic force microscopy (AFM) using the NT-MDT NTegra Maximus atomic force microscope from NT-MDT Spectrum Instruments (Moscow, Russia) with standard silicon cantilevers and a rigidity of 15 N·m^−1^ in tapping mode. AFM images were taken of the 2 membranes, with one photo at different membrane sites. The roughness measurement error was 20%.

### 2.7. Scanning Electron Microscopy

Scanning electron microscopy (SEM) was used to study the cross-sectional and surface morphology of the dense and supported PEBA-based membranes. A Zeiss AURIGA Laser (Carl Zeiss SMT, Oberhochen, Germany) was applied to carry out the experiment. 

### 2.8. Swelling Degree

The swelling degree (sorption) was studied in a water/isopropanol mixture, water, and isopropanol for dense PEBA and PEBA/Ho-1,3,5-H_3_btc membranes by using the gravimetric method at 25 °C. Each dense membrane was lowered into a water/isopropanol mixture, water, or isopropanol, and the weight of the membranes was checked regularly until a constant swelling weight was reached. To calculate the swelling degree (*S*), Equation (6) was used [52]:

(5) $$S=\frac{m_{s}-m_{o}}{m_{o}}\cdot 100\%$$

where *m_s_* (g) is the weight of the swollen dense membrane, and *m_o_* (g) is the initial weight of the dry dense membrane.

## 3. Results and Discussion

This section is divided into three subsections:

- “Section 3.1” focuses on the development of the dense PEBA and PEBA/Ho-1,3,5-H_3_btc membranes; the study of their transport is presented in “Section 3.1.1” and their structure and physicochemical properties are discussed in “Section 3.1.2”;
- “Section 3.2” focuses on the development of the supported PEBA and PEBA/Ho-1,3,5-H_3_btc membranes; the study of their transport is presented in “Section 3.2.1” and their physicochemical properties are discussed in “Section 3.2.2”;
- “Section 3.3” is dedicated to the comparison of the performance of the developed membranes with PEBA-based membranes from literature data.

### 3.1. The Development and Investigation of Dense PEBA and PEBA/Ho-1,3,5-H_3_btc Membranes

#### 3.1.1. Pervaporation Performance of Dense PEBA and PEBA/Ho-1,3,5-H_3_btc Membranes

**Figure 1:**
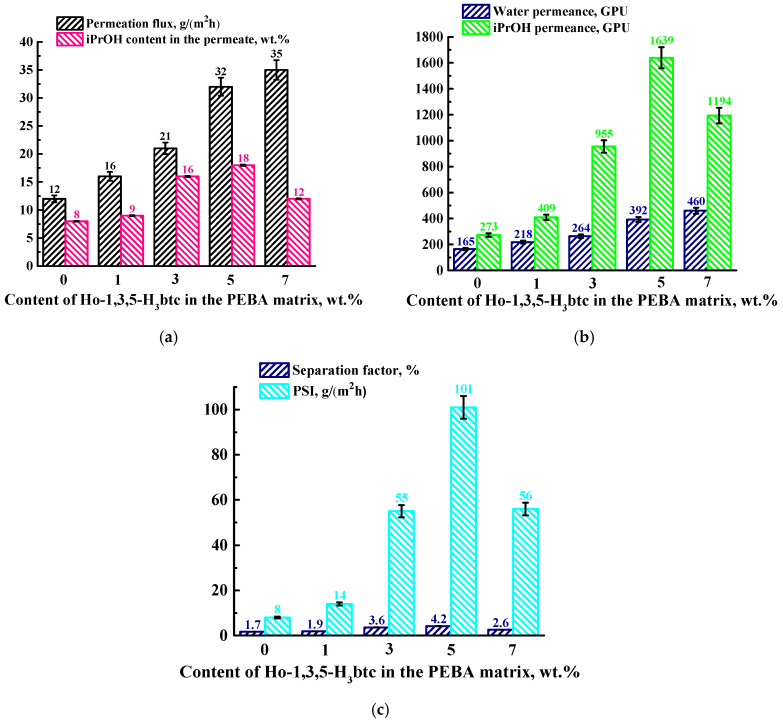
The dependence of (**a**) permeation flux, the isopropanol content in the permeate, (**b**) component permeance, and (**c**) separation factor and PSI on the Ho-1,3,5-H_3_btc content in the PEBA matrix for pervaporation separation of a water (95%)/iPrOH (5%) mixture.

In order to select the optimal concentration of the modifier, Ho-1,3,5-H_3_btc from 1 to 7 wt.% was introduced into the PEBA matrix. Figure 1 shows the transport properties (permeation flux, isopropanol content in permeate, separation factor, PSI, water, and isopropanol permeances) of the dense membranes based on PEBA and PEBA/Ho-1,3,5-H_3_btc (1–7 wt.%) for pervaporation separation of a water (95%)/iPrOH (5%) mixture.

It was found that the introduction of Ho-1,3,5-H_3_btc up to 7 wt.% led to an increase in permeation flux to 35 g/(m^2^h). The introduction of needle-shaped Ho-1,3,5-H_3_btc with a highly porous structure into the PEBA matrix led to morphological changes in the membrane during the modification process, namely the formation of “plastic deformations” in the inner structure and increased surface roughness (confirmed by SEM and AFM). This led to a greater effective active contact surface with increased sorption center numbers, causing more membrane swelling in the feed (confirmed by swelling degree data), which consequently led to an increase in permeability [56,57,58,59]. With the introduction of up to 5 wt.% Ho-1,3,5-H_3_btc into the PEBA matrix, a rise in isopropanol content in the permeate was noted to 18 wt.%, which could be associated with the hydrophobization of the membrane surface during the modification process [50], which is more attractive for isopropanol penetration. A further increase in the Ho-1,3,5-H_3_btc content in the PEBA matrix to 7 wt.% resulted in a decrease in the isopropanol content in the permeate to 12 wt.% compared to the PEBA-5 membrane. This effect may be caused by excessive swelling in isopropanol (confirmed by swelling degree data), which is embedded between the PEBA polymer chains, increasing the free volume and thereby facilitating the penetration of the second feed component—water [60]. Based on the component content in the permeate, the separation factor was also calculated to account for membrane selectivity, the values of which demonstrate the same dependence (Figure 1c).

A far more informative approach to present pervaporation data is through membrane permeances, as it directly reflects the intrinsic properties of the separation membranes [54]. It was shown that the PEBA-based membranes had more permeance for isopropanol than for water (Figure 1b). This may be due to the hydrophobic nature of PEBA, and the introduction of Ho-1,3,5-H_3_btc into the polymer matrix leads to the surface hydrophobization [50], causing an increase in the iPrOH permeance. The PSI calculated to account for the efficiency (Figure 1c) demonstrated that the PEBA-5 membrane (with 5 wt.% Ho-1,3,5-H_3_btc) had the optimal performance due to the highest value of both the separation factor and PSI.

Thus, the PEBA-5 membrane has the optimal pervaporation performance among developed dense membranes: the highest isopropanol content in the permeate with high permeation flux and the highest isopropanol permeance, separation factor, and PSI values, which demonstrate the efficiency of the separation of the water/isopropanol mixture. The introduction of 5 wt.% Ho-1,3,5-H_3_btc into the PEBA matrix was chosen as optimal for further membrane improvement through the development of supported membranes.

#### 3.1.2. Structure and Physicochemical Properties of Dense PEBA and PEBA/Ho-1,3,5-H_3_btc Membranes

**Figure 2:**
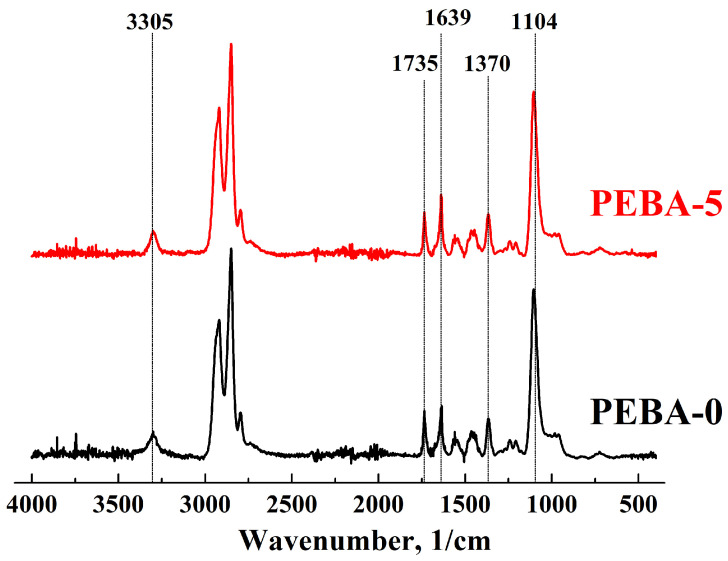
FTIR spectra of dense PEBA-0 and PEBA-5 membranes.

The structural characteristics of the PEBA and PEBA/Ho-MOFs membranes were studied by FTIR spectroscopy (Figure 2).

The FTIR spectra of the PEBA-0 membrane exhibited characteristic peaks attributed to various functional groups in the polyamide and polyether segments. The peak at 3305 cm^−1^ corresponds to the stretching vibration of N-H, the peak at 1735 cm^−1^ to the stretching vibration of C=O, the peak at 1639 cm^−1^ to the stretching vibration of H–N–C=O, and the peak at 1370 cm^−1^ to the stretching vibration of C–N groups in the polyamide segment [20,21]. The peak at 1104 cm^−1^ is assigned to the stretching vibration of the C-O-C group in the polyether segment [20,21,23,61]. For the modified PEBA-5 membrane, there were no new bands or band shifts in the FTIR spectrum. This suggests that the Ho-MOFs are physically blended with the PEBA matrix without forming chemical covalent bonds. Such interaction has been previously observed in studies devoted to the development of PEBA/MOF membranes [20,23].

**Figure 3:**
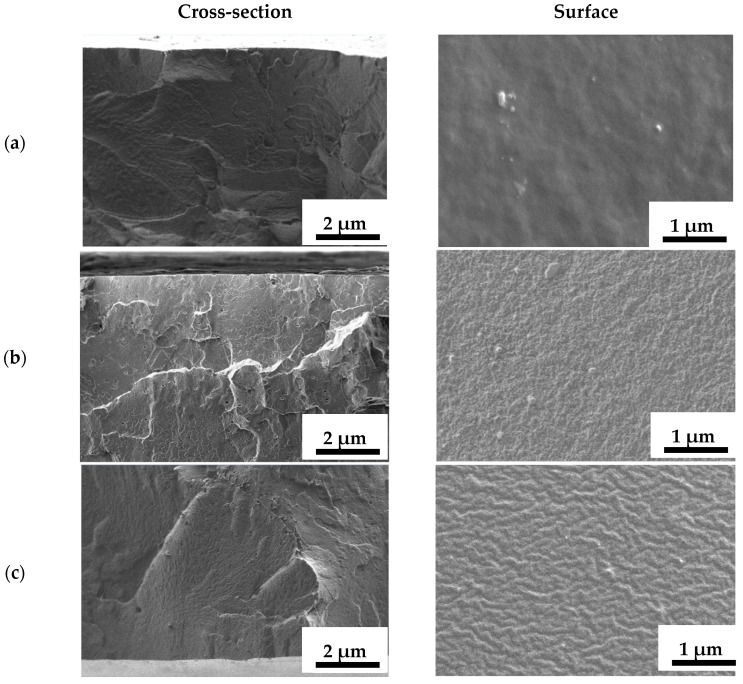

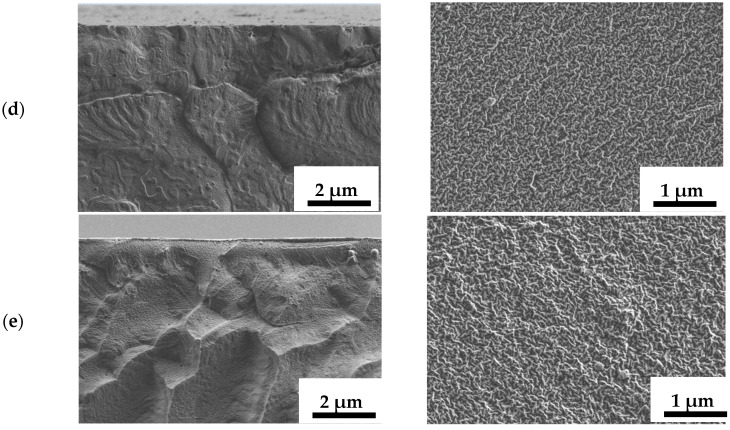
Cross-sectional and surface SEM micrographs of dense membranes based on PEBA and its composite with different Ho-1,3,5-H_3_btc contents: (**a**) PEBA-0, (**b**) PEBA-1, (**c**) PEBA-3, (**d**) PEBA-5, and (**e**) PEBA-7.

The morphology of the dense membranes based on PEBA and its composite with different contents of Ho-1,3,5-H_3_btc was studied by SEM (Figure 3).

The presented SEM micrographs of the pristine PEBA-0 membrane show a uniform surface structure with a rough and ribbed cross-sectional structure and the absence of visible defects (Figure 3a). The introduction of 1 wt.% Ho-1,3,5-H_3_btc leads to a slight change in the cross-section and surface structures, while a further increase in the modifier concentration strongly alters the internal morphology of the membranes, leading to the formation of gross “plastic deformations” on the cross-sectional structure, which may be due to the crystalline nature of the modifier. The surface of the modified membranes changes toward the formation of a more pronounced “comb-like” structure, which may be associated with the migration of needle-shaped Ho-1,3,5-H_3_btc to the surface of the membranes during their formation [62]. At the same time, the Ho-1,3,5-H_3_btc particles were not visible in the cross-sectional and surface structure of any of the modified membranes, indicating their uniform distribution in the polymer matrix and the absence of clusters and agglomerates of Ho-MOF. The absence of particle agglomerations would not create an obstacle to mass transport through modified membranes.

**Figure 4:**
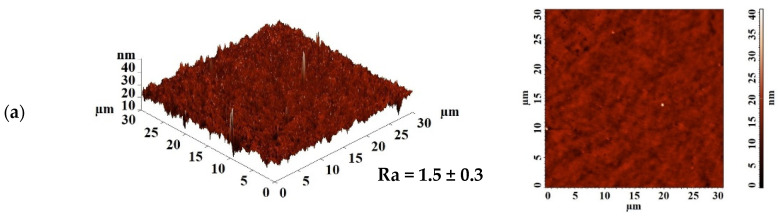

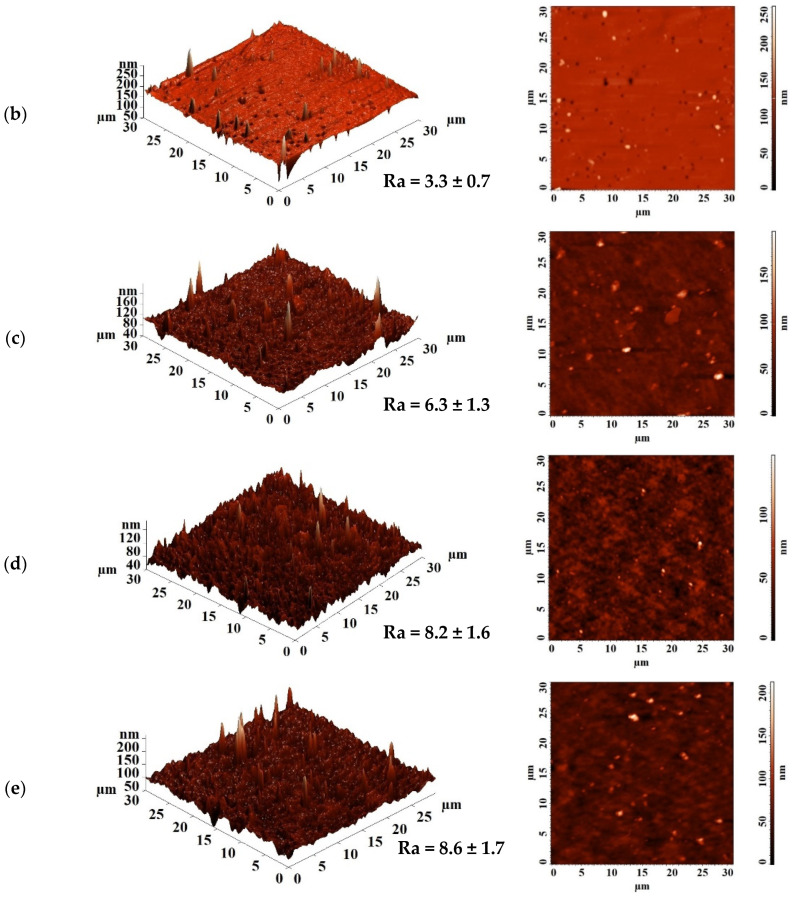
AFM images and average roughness values (Ra) of dense membranes based on PEBA and its composite with different Ho-1,3,5-H_3_btc contents: (**a**) PEBA-0, (**b**) PEBA-1, (**c**) PEBA-3, (**d**) PEBA-5, and (**e**) PEBA-7.

Since the first stage of pervaporation, according to the “solubility–diffusion” mechanism, is the sorption of the components of the separated mixture on the membrane surface, changes on the surface during modification should affect the membrane properties. The surface topology of membranes based on PEBA and the PEBA/Ho-MOF composite with different contents of Ho-1,3,5-H_3_btc was studied by AFM. AFM images with a scan size of 30 × 30 μm are shown in Figure 4. Based on AFM data, the average roughness (Ra) of the membrane surface was calculated and is also presented in Figure 4.

AFM data confirmed that the introduction of Ho-1,3,5-H_3_btc into the PEBA matrix resulted in an increase in the surface roughness of the membranes, which was consistent with the SEM data (Figure 3). The data in Figure 4 show that as the Ho-1,3,5-H_3_btc content in the PEBA matrix increases, there is an increase in the membrane average roughness. The PEBA-7 membrane has the highest average roughness (Ra = 8.6 nm) compared to the other membranes (also in agreement with SEM data (Figure 3e)), indicating that the largest number of Ho-1,3,5-H_3_btc particles migrated to the membrane surface. An increase in surface roughness provides a larger effective surface area for contact with the components of the feed, which is one of the factors leading to easier sorption and faster penetration of substances through the membrane. This leads to improved permeation flux of the modified membranes, which is consistent with the pervaporation data (Figure 1).

The swelling of the dense PEBA and PEBA/Ho-1,3,5-H3btc membranes was studied in a water/isopropanol (5 wt.% isopropanol) mixture, isopropanol, and water. Data on the swelling degree are presented in Table 2.

It was found that the developed dense membranes with an increasing content of Ho-1,3,5-H_3_btc increased their swelling ability in isopropanol, but practically did not swell in water and the water/isopropanol mixture (isopropanol 5 wt.%), since this mixture consists mainly of water. This was due to the introduction of a hydrophobic modifier into the PEBA matrix, leading to hydrophobization of the membrane surface (confirmed by the contact angle value increasing previously in [50]) and, as a result, an increase in the sorption of isopropanol on the membrane surface and improved permeation flux and isopropanol permeance for the modified membranes [32]. A high swelling degree in isopropanol was observed for all membranes, confirming the selectivity of the developed membranes toward alcohol. The highest degree of swelling in isopropanol was observed for the PEBA-7 membrane, which corresponded to the highest permeation flux among all modified membranes (Figure 1).

### 3.2. The Development and Investigation of the Supported PEBA and PEBA/Ho-1,3,5-H_3_btc Membranes

#### 3.2.1. Pervaporation Performance of Supported PEBA and PEBA/Ho-1,3,5-H_3_btc Membranes

**Figure 5:**
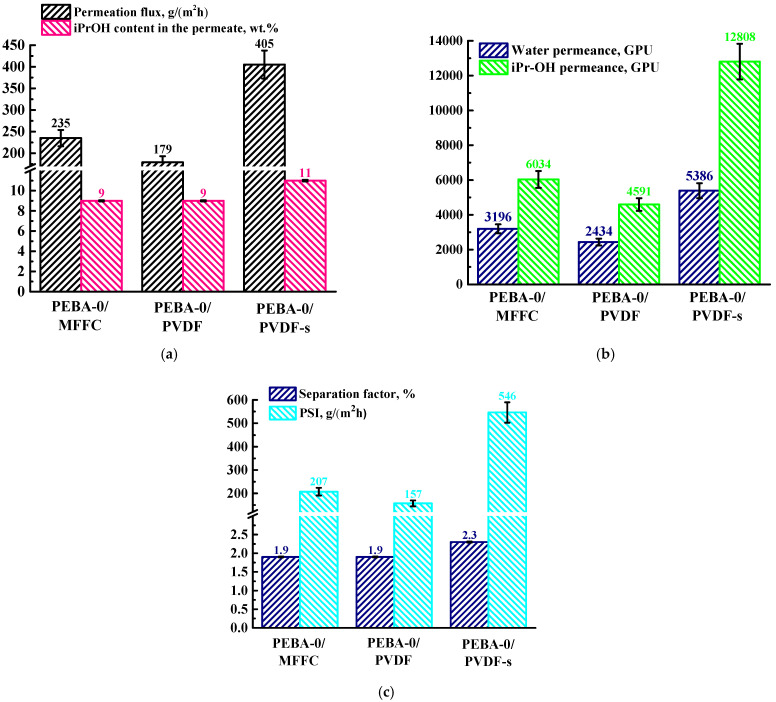
The (**a**) permeation flux, the isopropanol content in the permeate, (**b**) the components’ permeance, and (**c**) the separation factor and PSI for the unmodified supported membranes for pervaporation separation of a water (95%)/iPrOH (5%) mixture.

In order to increase the permeation flux of the dense membranes developed, supported membranes were developed, in which a thin non-porous polymer layer was applied to various porous polymer substrates. The type of polymer substrate, its porosity, and its nature can significantly influence the transport properties of the supported membrane [63,64,65,66]. Therefore, in this study, the influence of three porous substrates on the properties of the supported membranes was studied. A commercial porous membrane based on fluoroplastic F-42L applied to a non-woven support of thermally bonded polyester fibers (MFFC) and PVDF-based membranes prepared by using the phase inversion method on a glass (PVDF) and a polyester support (PVDF-s) were used as porous substrates for the development of supported PEBA membranes. Figure 5 shows the transport properties of the PEBA membranes supported on various substrates (MFFC, PVDF, and PVDF-s) during the separation of a water/isopropanol mixture (5 wt.% isopropanol).

It was found that the development of supported PEBA-based membranes led to an increase in permeation flux (20 times for the PEBA-0/MFFC membrane, 15 times for the PEBA-0/PVDF, and 34 times for the PEBA-0/PVFD-s membrane) while maintaining high isopropanol content in the permeate compared to the pristine dense PEBA-0 membrane (Figure 1). Based on the data obtained (Figure 5), it is clear that the supported PEBA-0/PVDF-s membrane has optimal transport characteristics (permeation flux of 405 g/(m^2^h), 11 wt.% isopropanol content in the permeate), which is also confirmed by the calculated data on the separation factor (2.3%), PSI (546 g/(m^2^h)), and isopropanol permeance (12,808 GPU). The highest transport characteristics of the PEBA-0/PVDF-s membrane are associated with the formation of the smallest selective layer (confirmed by SEM). It is also worth noting that the parameters of the PEBA-0/PVDF-s membrane were higher compared to the PEBA-0/PVDF membrane. The use of a non-woven support prevents shrinkage of the PVDF substrate, resulting in a more porous and regular structure that has less effect on the mass transfer of components through the supported membrane [67]. Meanwhile, the formation of a porous membrane (in this case, a PVDF substrate) without a support results in smaller pores due to polymer shrinkage [67].

**Figure 6:**
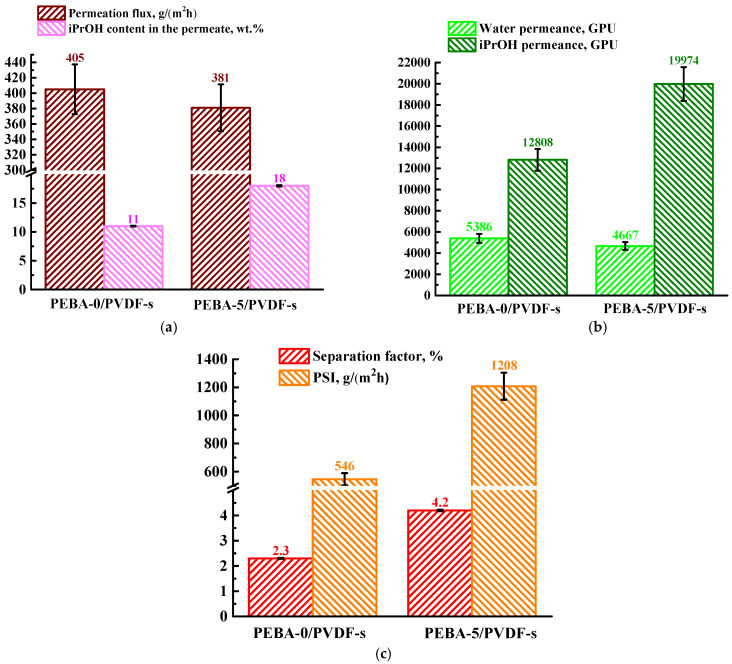
The values of (**a**) permeation flux, isopropanol content in the permeate, (**b**) the components’ permeance, and (**c**) the separation factor and PSI for the supported PEBA-0/PVDF-s and PEBA-5/PVDF-s membranes for pervaporation separation of a water (95%)/iPrOH (5%) mixture.

Thus, the porous PVFD-s substrate (prepared with a non-woven support) was chosen as optimal for creating supported membranes via deposition of a dense PEBA-based layer modified with 5 wt.% Ho-1,3,5-H_3_btc (PEBA-5/PVDF-s membrane). The transport properties of this developed membrane were also investigated by pervaporation separation of a water/isopropanol mixture (5 wt.% iPrOH) (Figure 6). For comparison, Figure 6 also shows data for the unmodified PEBA-0/PVDF-s membrane.

It was found that the introduction of Ho-1,3,5-H_3_btc led to a slight decrease in permeation flux from 405 (for PEBA-0/PVDF-s membrane) to 381 g/(m^2^h) (for PEBA-5/PVDF-s membrane), which could be due to an increase in the thickness of the modified selective PEBA/Ho-1,3,5-H_3_btc (5%) layer (confirmed by SEM). Previously, an increase in the thickness of the selective layer of the membranes was also observed when MOFs were introduced into the polymer matrix [68,69]. Also, the introduction of Ho-1,3,5-H_3_btc into the supported membrane led to an increase in the isopropanol content in the permeate, the isopropanol permeance, and the PSI values due to its hydrophobic nature and porous structure, leading to membrane surface roughness (confirmed by AFM data) and hydrophobization [50], which also increased swelling in isopropanol (confirmed by swelling degree, Table 3).

**Figure 7:**
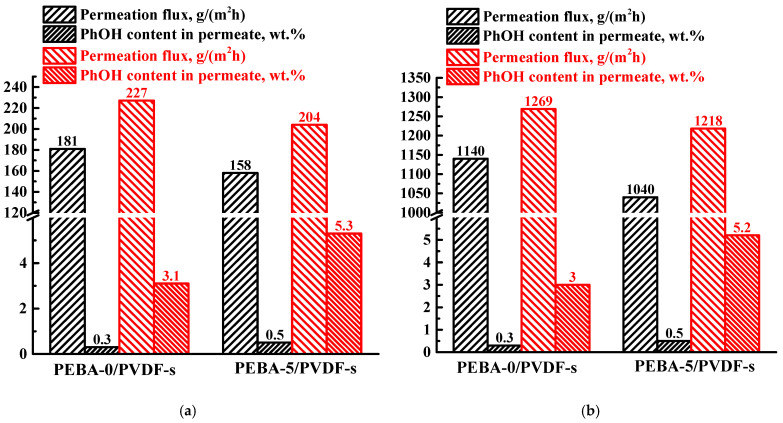
The permeation flux and phenol content in the permeate for the supported PEBA-0/PVDF-s and PEBA-5/PVDF-s membranes for pervaporation separation of a water/phenol mixture of 0.1 wt.% (black) and 1 wt.% phenol (red) at (**a**) 22 °C and (**b**) 50 °C.

Also, PEBA-based membranes are most often used for phenol removal. So, the supported PEBA-0/PVDF-s and PEBA-5/PVDF-s membranes were studied in the separation of water/phenol mixtures with a phenol content of 0.1 and 1 wt.% at 22 and 50 °C. The obtained transport characteristics are presented in Figure 7.

It was found that when Ho-1,3,5-H_3_btc was introduced into the PEBA matrix, the phenol content in the permeate increased, while the permeation flux slightly decreased, as in the case of pervaporation separation of the water/isopropanol mixture (Figure 6). The decrease in permeation flux can also be associated with an increase in the thickness of the selective layer of the modified membrane. The increase in selectivity toward phenol may also be due to surface hydrophobization of the Ho-1,3,5-H_3_btc-modified membrane [50], leading to increased sorption of the more hydrophobic component (phenol) on the membrane surface and its swelling. Previous studies [21,22] have also observed an increase in the water contact angle upon the introduction of MOFs, leading to an increase in selectivity toward phenol. With an increase in the phenol content in the feed from 0.1 to 1 wt.%, an increase in permeation flux was observed for both PEBA-0/PVDF-s and PEBA-5/PVDF-s membranes, as for pervaporation both at 22 and 50 °C. Increasing the temperature from 22 to 50 °C led to an increase in the permeation flux, maintaining selectivity. This effect may be due to the fact that with the temperature rise, the mobility of polymer chains increases, causing an increase in free volume between polymer chains, which results in improved component transfer through membranes. The same effect of temperature on permeation flux was previously noted for membranes based on polyimide synthesized from different dianhydrides during pervaporation of an ethanol–water mixture [70,71].

#### 3.2.2. Structure and Physicochemical Properties of Supported PEBA and PEBA/Ho-1,3,5-H3btc Membranes

**Figure 8:**
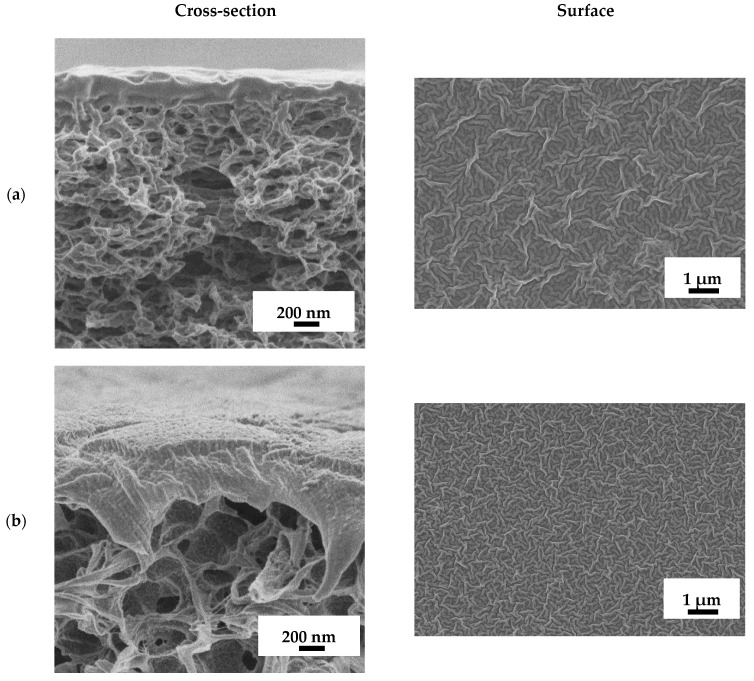

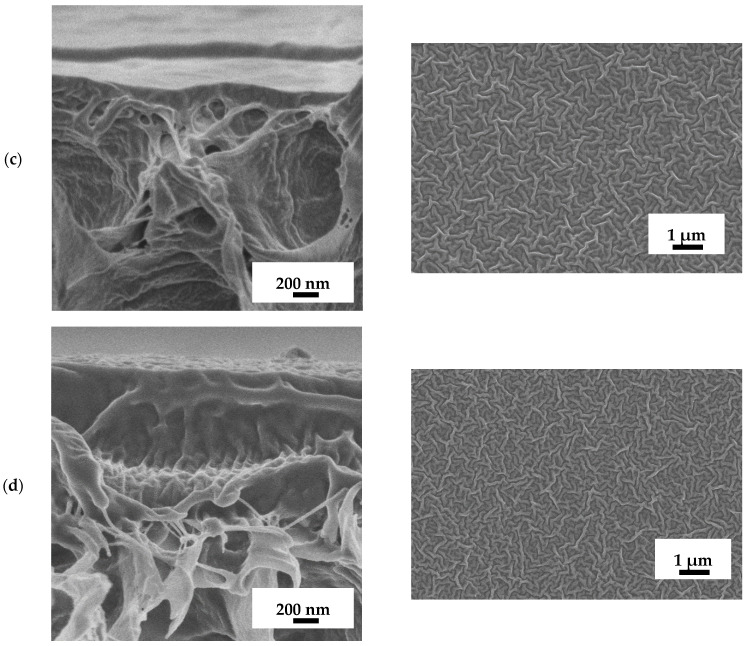
SEM micrographs of supported membranes: (**a**) PEBA-0/MFFC, (**b**) PEBA-0/PVDF, (**c**) PEBA-0/PVDF-s, and (**d**) PEBA-5/PVDF-s.

The use of substrates of different materials for the development of supported PEBA membranes may result in the formation of a selective layer with various thicknesses, structures and roughness. Although it is generally accepted that the substrate or support (such as a non-woven polyester support) does not affect the mass transfer of components across the supported membrane during pervaporation, it may lead to the formation of the selective polymer layer with different structural properties and transport characteristics [63,64,65,66]. Therefore, the characterization of supported membranes is important to explain the performance of the obtained PEBA-based membranes. The inner and surface structure of the developed supported PEBA-based and PEBA-5/PVDF-s membranes was studied by SEM. Cross-section and surface micrographs are presented in Figure 8.

It was found that the use of different substrates resulted in the formation of a selective layer of different thicknesses. Thus, when using the commercial porous membrane MFFC, the selective layer based on PEBA with a thickness of ~250 nm was formed on the surface of the porous substrate. The use of the porous PVDF-based membranes prepared on different supports (glass (PVDF) or polyester support (PVDF-s)) resulted in the formation of PEBA-based selective layers of different thicknesses for the supported membranes: ~520 nm for PEBA-0/PVDF and ~150 nm for PEBA-0/PVDF-s membranes. Thus, the PEBA-0/PVDF membrane with the greatest thickness of the selective layer among the unmodified supported membranes had the lowest values of permeation flux (Figure 5). SEM micrographs of the surface show a similar surface structure of the unmodified supported membranes (Figure 8a–c). The introduction of Ho-MOF led to an increase in the thickness of the selective layer to ~970 nm, which resulted in a slightly decreased permeation flux (Figure 5, Figure 6 and Figure 7) of the modified supported PEBA-5/PVDF-s membrane compared to the PEBA-0/PVDF-s membrane.

**Figure 9:**
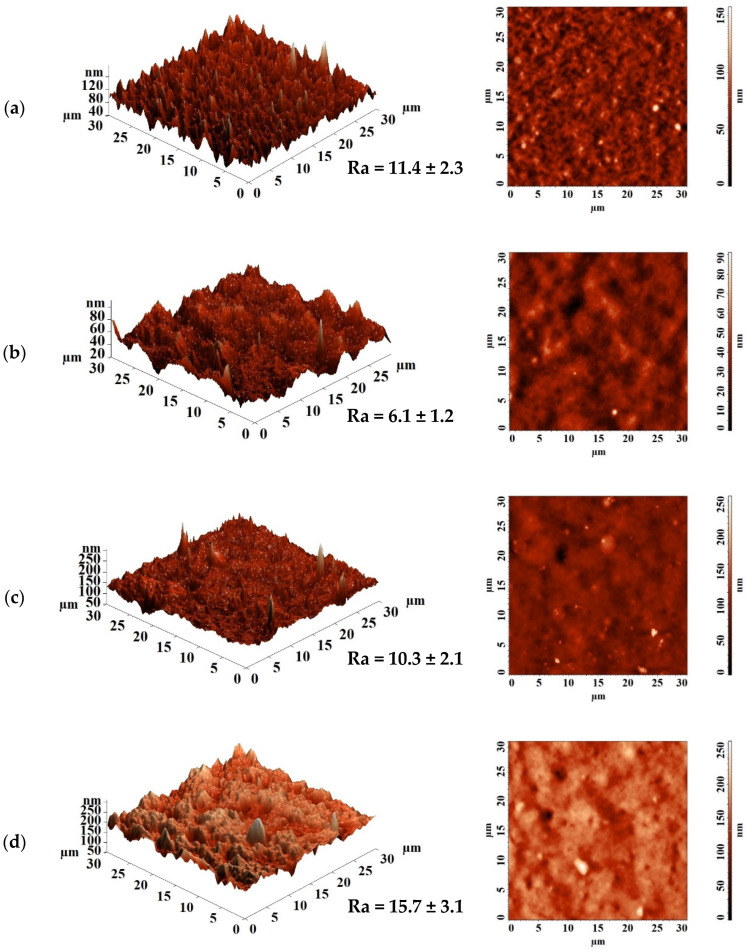
AFM images and average roughness values (Ra) for supported membranes: (**a**) PEBA-0/MFFC, (**b**) PEBA-0/PVDF, (**c**) PEBA-0/PVDF-s, and (**d**) PEBA-5/PVDF-s.

The surface roughness of the developed supported unmodified PEBA and PEBA-5/PVDF-s membranes was studied by AFM. AFM images with a scanning size of 30 × 30 μm are shown in Figure 9. Based on AFM data, the average roughness (Ra) of the supported membrane surface was calculated and is also presented in Figure 9.

According to the data in Figure 9, it is evident that the deposition of the thin PEBA and PEBA/Ho-1,3,5-H_3_btc (5%) layers on porous substrates results in a significant increase in surface roughness compared to dense membranes (Figure 4). It may explained by the effect of using substrates to create a thin, dense selective layer that follows their irregularities [72]. An increase in surface roughness, when a thin selective layer was applied onto a porous substrate, was also observed in [73]. The surface parameter of the PEBA-5/PVDF-s membrane is significantly higher compared to the other supported membranes due to the presence of the Ho-1,3,5-H_3_btc modifier in the thin selective layer, leading to an increase in the sorption of isopropanol and phenol on the modified membrane surface and a rise in selectivity.

### 3.3. Comparison of Performance with PEBA-Based Membranes

The transport properties of the developed supported membranes PEBA-0/PVDF-s and PEBA-5/PVDF-s were compared with the literature data on PEBA-based membranes applied for the separation of water/phenol mixtures by pervaporation under comparable conditions to those in the present study. The comparison is presented in Table 3.

**Table 3:** The comparison of the transport properties of the PEBA-based membranes for the separation of water/phenol mixtures by pervaporation.

Membranes	T, °C	Phenol Content in the Feed, wt.%	Permeation Flux, g/(m^2^h)	Separation Factor	References
PEBA-5/PVDF-s	50	0.1	1040	5.0	This study
PEBA-0/PVDF-s	50	0.1	1140	3.0	This study
PEBA + polyurethane (1:1)	35	0.1	84.1	9.7	[74]
ZIF-71P/PEBA-21	50	0.1	911.1	18.96	[46]
ZIF-71K/PEBA-21	50	0.1	965.49	17.74	
40%Co-UMOFNs-PEBA/PVDF	70	0.1	420	45.49	[20]
PEBA + MAF-6 (7 wt.%)	80	0.1	3520	25.9	[22]
S-ZIF-8/PEBA/ZIF-8-8	80	0.2	198	114	[75]
PEBA/HZIF-8-10	80	0.2	247.7	80.89	[18]
PEBA-5/PVDF-s	50	1	1218	5.4	This study
PEBA-0/PVDF-s	50	1	1269	3.1	This study
S-ZIF-8/PEBA/ZIF-8-8	70	0.8	215.85	109.3	[75]
PEBA-2533	70	0.6–0.8	1100	23	[76]
PEBA-2533/ZIF-8 (10%)	70	0.8	1310	53	[77]

The data presented in Table 3 demonstrate that the supported PEBA-0/PVDF-s and PEBA-5/PVDF-s membranes have good transport properties, for pervaporation of the water/phenol mixture with both 0.1 and 1 wt.% of phenol in the feed. The higher permeation flux of some membranes reported in the literature can be caused by elevated temperature. Based on the obtained results, it can be concluded that the developed supported PEBA-0/PVDF-s and PEBA-5/PVDF-s membranes are promising for industrial applications of pervaporation.

## 4. Conclusions

In the present study, advanced pervaporation mixed-matrix membranes based on PEBA modified with an Ho-1,3,5-H_3_btc MOF were developed for the separation of water/isopropanol and water/phenol mixtures.

The introduction of Ho-1,3,5-H_3_btc from 1 to 7 wt.% in the dense PEBA membrane led to the increase in permeation flux and the isopropanol content in the permeate for pervaporation separation of a water (95%)/iPrOH (5%) mixture. It was associated with a rise in the membrane’s inner and surface structural roughness (confirmed by SEM and AFM) and surface hydrophobization (confirmed by swelling degree data) due to the introduction of hydrophobic highly porous needle-shaped Ho-1,3,5-H_3_btc. The dense PEBA-5 membrane (with 5 wt.% Ho-1,3,5-H_3_btc) had optimal pervaporation performance: a 3-times greater permeation flux compared to the PEBA-0 membrane, and the highest isopropanol permeance, separation factor, and PSI values among all membranes.

To increase the permeability of dense membranes and to study the influence of porous substrate selection, supported membranes from the pristine PEBA layer deposited on various porous substrates (commercial MFFC, developed PVDF without a non-woven support and PVDF-s with a non-woven support) were developed. These supported membranes demonstrated a more than 15-times higher permeation flux, while maintaining high isopropanol content in the permeate, compared to the dense PEBA-0 membrane. The porous PVFD-s substrate (prepared with a non-woven support) was chosen as optimal for further modification of its PEBA-based layer with 5 wt.% Ho-1,3,5-H_3_btc due to the highest performance of the supported membrane prepared on it.

The developed supported PEBA/Ho-1,3,5-H_3_btc (5%)/PVFD-s membrane demonstrated a 32-times improved permeation flux and a 10% higher isopropanol content in the permeate compared to the dense PEBA membrane. Also, its performance was evaluated for the separation of water/phenol mixtures (0.1 and 1 wt.% phenol) at 22 and 50 °C, where it demonstrated improved properties compared to the unmodified supported membrane and its potential for the extraction of phenol.

## Figures and Tables

**Table 1:** Developed dense and supported membranes based on PEBA and PEBA/Ho-1,3,5-H_3_btc composites.

Membrane	Type	Content of Ho-1,3,5-H_3_btc, wt.%	Support
PEBA-0	dense	0	-
PEBA-1	dense	1	-
PEBA-3	dense	3	-
PEBA-5	dense	5	-
PEBA-7	dense	7	-
PEBA-0/MFFC	supported	0	MFFC
PEBA-0/PVDF	supported	0	PVDF
PEBA-0/PVDF-s	supported	0	PVDF-s
PEBA-5/PVDF-s	supported	5	PVDF-s

**Table 2:** The swelling degree of the dense PEBA and PEBA/Ho-1,3,5-H_3_btc membranes in a water/isopropanol (5 wt.% isopropanol) mixture, isopropanol, and water.

Membrane	Swelling Degree, %		
	Water	Isopropanol	Water (95%)/Isopropanol (5%)
PEBA-0	2 ± 0.2	100 ± 10	4 ± 0.4
PEBA-1	4 ± 0.4	132 ± 13	6 ± 0.6
PEBA-3	5 ± 0.4	141 ± 14	8 ± 0.8
PEBA-5	6 ± 0.6	150 ± 15	8 ± 0.8
PEBA-7	8 ± 0.7	170 ± 17	10 ± 1

## Data Availability

The original contributions presented in this study are included in the article. Further inquiries can be directed to the corresponding authors.
